# From Kerr to Heisenberg

**DOI:** 10.3390/e23030315

**Published:** 2021-03-07

**Authors:** Angelo Tartaglia, Matteo Luca Ruggiero

**Affiliations:** 1 INAF-OATo, Via Osservatorio 20, 10025 Pino Torinese, Italy; 2 Department of Applied Science and Technology, Politecnico di Torino, 10129 Torino, Italy; matteo.ruggiero@polito.it; 3 INFN—National Laboratories of Legnaro, Viale dell’Università 2, 35020 Legnaro, Italy

**Keywords:** gravito-magnetism, quantum mechanics, Heisenberg principle

## Abstract

In this paper, we consider the space-time of a charged mass endowed with an angular momentum. The geometry is described by the exact Kerr–Newman solution of the Einstein equations. The peculiar symmetry, though exact, is usually described in terms of the gravito-magnetic field originated by the angular momentum of the source. A typical product of this geometry is represented by the generalized Sagnac effect. We write down the explicit form for the right/left asymmetry of the times of flight of two counter-rotating light beams along a circular trajectory. Letting the circle shrink to the origin the asymmetry stays finite. Furthermore it becomes independent both from the charge of the source (then its electromagnetic field) and from Newton’s constant: it is then associated only to the symmetry produced by the gravitomagnetic field. When introducing, for the source, the spin of a Fermion, the lowest limit of the Heisenberg uncertainty formula for energy and time appears.

## 1. Introduction

The one century long story of the parallel development of the two main physical theories of the 20th century is far from an end and continues to puzzle our understanding with being both brilliantly successful in their proper domain and in the same time diverging or even being in conflict in the areas where they are obliged to cohabit. We are obviously referring to General Relativity (GR) on one side and Quantum Mechanics (QM) on the other. We will not enter here the intricate problem usually called “quantization of gravity”. An important portion of the theoretical physicists community continues to work on it, mostly with an intellectual prejudice on the fact that space-time must be quantizable as if it were a field more or less like others and so confusing the container (space-time) with the content (fields).

We will simply try to evidence in the following a peculiar case where a link is directly manifested between GR and QM, without the need to introduce new tools and modifications of one or the other theory.

One of the weakest effects of the gravitational interaction as described by GR has been dubbed gravitomagnetism. It depends on the off-diagonal time-space terms of the metric tensor, $g_{0i}$. The usual conventions are applied for notation: indices running from 0 to 3 are denoted by Greek letters; space indices running from 1 to 3 are represented by Latin letters; three-dimensional vectors are represented by bold letters. The Einstein summation rule of equal co- and contra-variant indices always holds). We will then concentrate on the travel times of test particles moving with locally constant velocity along trajectories closed in space. “Closed in space” is not an absolute statement since closedness in three space dimensions depends on the choice of the reference frame; however there is something which does not depend on such choice. When either the observer or the source of gravity or both are rotating the times of travel of test particles moving with the same local speed in opposite directions along the same closed trajectory (closed in the reference frame of the given observer) turn out to be different. The simplest and typical case is the one known as the Sagnac effect [1]: a rotating observer, in flat (Minkowski) space-time, sending light signals along a closed contour in opposite directions (only excluding the case in which the contour is contained in a plane parallel to the rotation axis) finds that the times of flight are different even though the path is the same. The difference is in terms of proper time of the observer, i.e., it is (proportional to) the length of a space-time interval, which means that it does not depend on the reference frame and stays there for any observer [2]. In flat space-time a rotating observer is of course not inertial and the closed path of the test particles (be they light beams or locally isotachic objects) must be obtained using appropriate physical devices. The time of flight (tof) asymmetry may be described using a different narration by different observers, but the result is exactly the same for everybody. In a rotating reference frame we have non-zero off diagonal terms in the metric, whilst in the frame of an inertial observer the metric is diagonal: the asymmetry is the same for both. This is however Special Relativity: we are now interested in GR.

Our case considers the space-time of a spinning source of gravity, i.e., in geometrical terms, of curvature. Now we have in general to do with a metric with non-null $g_{0i}$ terms. It is always true that we may locally diagonalize the metric (this happens because, apart from singularities, there always is a local tangent flat space-time); the diagonalization is however not contextually possible everywhere. The nickname “gravitomagnetism” (GM) is, as known, due to the fact that, in weak field approximation, the Einstein equations in vacuo assume a form practically coinciding (but for a factor of 2) with the classical Maxwell equations of electromagnetism (EM) and $g_{0i}$ may, in the same approximation, be treated as the components of a three-dimensional vector potential in analogy with EM [3,4,5,6,7,8,9,10,11].

Here, however, we shall use no approximation though continuing to use the GM terminology. In this framework we shall evidence the somehow surprising connection between GR and QM.

## 2. Generalized Sagnac Effect

We consider the line-element in the form

(1) $$ds^{2}=g_{00}c^{2}dt^{2}+2g_{0i}cdtdx^{i}+g_{ij}dx^{i}dx^{j}$$

where $g_{\mu \nu }=g_{\mu \nu }\left(x\right)$, hence the space-time metric does not depend on time. The above metric is quite general in its form and, in particular, it is said to be non time-orthogonal, because $g_{0i}\neq 0$. These off-diagonal terms are related to the rotation of the sources of the gravitational field and on the rotational features of the reference frame: in the case of a rotating frame in flat space-time, they depend on the rotation rate; more in general, they express the rotation rate of the frame with respect to a Fermi–Walker tetrad (see, e.g., [12]).

We want to calculate the asymmetry in the propagation times of two signals in the space-time described by the line-element (1); this asymmetry is the so-called Sagnac time delay (see, e.g., [13,14]). We consider two messengers (massive or massless particles) simultaneously emitted at a given location: they propagate in opposite directions along the same path and reach the emission point at different times, thus evidencing an asymmetry. In doing so, we need to impose some conditions to say that the particles propagating in the two opposite directions are identical but differ only for the direction of propagation: this is naively related to their speed. However, the coordinate speed $w^{i}=\frac{dx^{i}}{dt}$ has not a direct physical meaning. If we want to give an operational meaning (i.e., in terms of observable quantities) to the speed of a particle, we may proceed as follows. Let us consider a set of observers located along the given closed trajectory and mutually at rest. One of them is the main observer *O* from which the travel of the oppositely moving particles starts. For any observer of the group (let us call it O1) it is possible to introduce an inertial frame, relative to which O1 is at rest: this is the so-called Locally Co-Moving Inertial Frame (LCIF). In this frame, the proper element of distance dσ and time dT can be defined in terms of the metric elements and coordinates intervals and the speed of the particle when passing by O1 is well defined. It is (see [13,15])

(2) $$d\sigma^{}=\sqrt{\gamma_{ij}dx^{i}dx^{j}},\;dT^{}=-\frac{1}{c}\frac{g_{\mu 0}}{\sqrt{-g_{00}}}dx^{\mu }$$

where $\gamma_{ij}=\left(g_{ij}-\frac{g_{i0}g_{j0}}{g_{00}}\right)$. If we use these expressions the line-element (1) takes the Minkowskian form

(3) $$ds^{2}=d\sigma^{2}-c^{2}dT^{2}$$

Consequently, for an observer at rest in this LCIF the speed of a moving particle is $v=\frac{d\sigma }{dT}$, that is the ratio between the proper element of distance dσ traveled in a proper time interval dT. This speed is well defined from an operational viewpoint and, as we are going to show, it is useful to define a natural condition on the properties of the two counter propagating particles.

On substituting in (3), we get

(4) $$ds^{2}=\left(1-\frac{c^{2}}{v^{2}}\right)d\sigma^{2}=\left(1-\frac{c^{2}}{v^{2}}\right)\gamma_{ij}dx^{i}dx^{j}$$

and from (1) we obtain

(5) $$\left(1-\frac{c^{2}}{v^{2}}\right)\gamma_{ij}dx^{i}dx^{j}=g_{00}c^{2}dt^{2}+2g_{0i}cdtdx^{i}+g_{ij}dx^{i}dx^{j}$$

Equation (5) can be solved for the coordinate time interval dt; to this end, we introduce β≐v/c. Notice that for light-like particles, on setting $ds^{2}=0$, we get β=1, in agreement with the second postulate of special relativity, and the left hand side of Equation (5) is equal to zero. On using the definition of $\gamma_{ij}$, Equation (5) now reads

(6) $$0=g_{00}c^{2}dt^{2}+2g_{0i}cdtdx^{i}+\left(\frac{1}{\beta^{2}}\gamma_{ij}+\frac{g_{i0}g_{j0}}{g_{00}}\right)dx^{i}dx^{j}$$

from which we obtain the two solutions

(7) $$dt_{\pm }=\frac{1}{|g_{00}|c}\left(g_{0i}dx^{i}\pm \frac{1}{\beta }\sqrt{|g_{00}|\gamma_{ij}dx^{i}dx^{j}}\right)=\frac{1}{|g_{00}|c}\left(g_{0i}dx^{i}\pm \sqrt{|g_{00}|}\frac{\left|d\sigma \right|}{\beta }\right)$$

In the above result, we may distinguish two contributions to the propagation time: the first is due to the synchronization of distant clocks (see, e.g., [15]) in the metric (1); the second describes the time occurring to cover the (proper) distance |dσ| with the local speed v=βc. In particular, we see that the second contribution in (7) does not depend on the propagation direction, since it does not change when $dx^{i}\to -dx^{i}$ if we assume that the speed *v* (or equivalently β) is a function only of the position along the path; the case v=constant along the path is a particular sub-case. This amounts to saying that, in any LCIF along the path, particles have the same velocity *v* in opposite directions. On the contrary, the other contribution depends on the propagation direction. Once the propagation path is known, (7) can be integrated to obtain the coordinate time interval (remember that we are interested in the future oriented branch of the light cone). Then, the asymmetry in the propagation times is given by

(8) $$\Delta t=\frac{2}{c}\oint_{\ell }\frac{g_{0i}dx^{i}}{|g_{00}|}=-\frac{2}{c}\oint_{\ell }\frac{g_{0i}dx^{i}}{g_{00}}$$

Of course the particles take different times for propagating along the path, depending on their speed, but what we have just shown is that the difference between these times is always given by Equation (8), in any stationary space-time, and for arbitrary paths, both for matter and light particles, independently of their physical nature.

## 3. Geometric Approach to the Gravitomagnetic Clock Effect

A variant of the generalized Sagnac effect is the so called GM clock effect [16]: Two identical clocks freely falling along a spatially closed orbit, in opposite directions, show a growing synchronization defect each time they cross each other. Whenever the orbit is circular (then the motion is uniform) the clock effect may also be described in terms of Minkowski geometry on the flat 1+1 dimensional surface of a cylinder (see Tartaglia [17]).

Suppose that two identical clocks move in opposite directions along the same circular orbit at radius r=R. At proper time τ=0 they cross at some point of the orbit and turn out to be synchronous there. The orbital speeds are in general different; let us call $\omega_{+}$ the one of the corotating clock (same sense as the central mass) and $\omega_{-}$ the one of the counter-rotating clock; it is $\omega_{+}>0$ and $\omega_{-}<0$. The proper times shown by the two clocks, when they meet for the second time, will in general be different. Under the assumed conditions the equations of motion along the orbit are plainly linear and, as seen by a distant observer, may be written

(9) $$\phi_{+}=\omega_{+}t;\;\phi_{-}=\omega_{-}t$$

The meeting point *C* satisfies the obvious condition $\phi_{C+}-\phi_{C-}=2\pi$ at the same coordinate time $t=t_{C}$. It is:

(10) $$t_{C}=\frac{2\pi }{\omega_{+}-\omega_{-}}$$

This condition, using Minkowski geometry (in practice the calculation is the one for the length of a side of a scalene triangle, given the other two and in presence of a Lorentzian signature) leads to the proper times of the two clocks [17]:

(11) $$\left\{\begin{array}{c} \tau_{C+}=t_{C}\sqrt{g_{00}+2g_{0\phi }\omega_{+}+g_{\phi \phi }\omega_{+}^{2}}=\frac{2\pi }{\omega_{+}-\omega_{-}}\sqrt{g_{00}+2g_{0\phi }\omega_{+}+g_{\phi \phi }\omega_{+}^{2}}\\ \tau_{C-}=t_{C}\sqrt{g_{00}+2g_{0\phi }\omega_{-}+g_{\phi \phi }\omega_{-}^{2}}=\frac{2\pi }{\omega_{+}-\omega_{-}}\sqrt{g_{00}+2g_{0\phi }\omega_{-}+g_{\phi \phi }\omega_{-}^{2}} \end{array}\right.$$

These results are exact and, as far as it is $\omega_{+}\neq \omega_{-}$ it is also $\tau_{C+}\neq \tau_{C-}$. In the case of freely orbiting test masses, the two orbital velocities appearing in the $\tau_{C\pm }$ may be determined solving the equations for the geodesics of the considered space-time and imposing the conditions assumed for the problem.

All the above corresponds to the strict definition of the clock effect; however another case, recalling the generalized Sagnac effect, is the difference of the tof for the two clocks coming back where observer *O* is. Now the spanned angle is in any case 2π if we assume the local observer to be at rest with the distant one. If the angular velocities in the two senses are different we expect also the periods $T_{+}$ and $T_{-}$ to be different:

$$T_{\pm }=\frac{2\pi }{\left|\omega_{\pm }\right|}$$

Passing to the corresponding proper times of the two “messengers” (again using the geometrical approach) it is

$$\tau_{O\pm }=\sqrt{g_{00}T_{\pm }^{2}\pm 4\frac{\pi }{c}g_{0\phi }T_{\pm }+4\frac{\pi^{2}}{c^{2}}g_{\phi \phi }^{2}}$$

The time asymmetry $\Delta \tau_{O}=\tau_{O+}-\tau_{O-}$ will be obtained comparing the readings on both clocks when they come back to *O*, now at different times. In the case of light we have to do with null world-lines and a difference is found looking at the clock of the observer in *O* (or considering the beat frequency in a ring laser at rest with *O* and coinciding with the closed contour travelled by the light beams [18]). The condition is now:

$$g_{00}T^{2}+4\frac{\pi }{c}g_{0\phi }T+4\frac{\pi^{2}}{c^{2}}g_{\phi \phi }=0$$

whence

$$T_{\pm }=\mp \frac{2\pi }{c}\frac{g_{0\phi }}{g_{00}}+\frac{2\pi }{c}\frac{\sqrt{g_{0\phi }^{2}-g_{00}g_{\phi \phi }}}{g_{00}}$$

The tof asymmetry is:

(12) $$\Delta T=T_{-}-T_{+}=\frac{4\pi }{c}\frac{g_{0\phi }}{g_{00}}$$

coinciding in practice with (8).

All this is in terms of global coordinates; if we wish to know what is locally measured by observer *O* we have to multiply by $\sqrt{g_{00}}$ and we obtain

$$\Delta T_{O}=\frac{4\pi }{c}\frac{g_{0\phi }}{\sqrt{g_{00}}}$$

The situation is a bit more complicate if *O* is moving, and of course it is moving if it is in free orbital motion around the central mass. Assuming again that the trajectory is a circle, let us call Ω the orbital velocity with respect to the distant observer. Projecting $\Delta T_{O}$ onto the worldline of the moving observer yields:

(13) $$\Delta T_{\Omega }=\Delta T\frac{\sqrt{g_{00}}+\frac{g_{0\phi }}{\sqrt{g_{00}}}\frac{\Omega }{c}}{\sqrt{g_{00}+2g_{0\phi }\frac{\Omega }{c}+g_{\phi \phi }\frac{\Omega^{2}}{c^{2}}}}=\frac{4\pi }{c}\frac{g_{0\phi }}{g_{00}}\frac{\sqrt{g_{00}}+\frac{g_{0\phi }}{\sqrt{g_{00}}}\frac{\Omega }{c}}{\sqrt{g_{00}+2g_{0\phi }\frac{\Omega }{c}+g_{\phi \phi }\frac{\Omega^{2}}{c^{2}}}}$$

Given the conditions we have chosen, Ω is of course not a free parameter. Since the metric tensor does not depend on ϕ and *t* we have two constants of motion: *E* (energy per unit mass) and *L* (angular momentum per unit mass). The time and ϕ components of the four-velocity of a test mass, *u*, are:

$$\left\{\begin{array}{c} u^{0}=\frac{Eg_{\phi \phi }-Lg_{0\phi }}{g_{00}g_{\phi \phi }-g_{0\phi }^{2}}\\ u^{\phi }=\frac{Lg_{00}-Eg_{0\phi }}{g_{00}g_{\phi \phi }-g_{0\phi }^{2}} \end{array}\right.$$

Then we obtain, for any free fall, including circular orbits:

(14) $$\frac{\Omega }{c}=\frac{u^{\phi }}{u^{0}}=\frac{d\phi }{cdt}=\frac{Lg_{00}-Eg_{0\phi }}{Eg_{\phi \phi }-Lg_{0\phi }}$$

Introducing (14) into (13) we get:

(15) $$\Delta T_{\Omega }=\frac{4\pi }{c}\frac{g_{0\phi }}{{\left(g_{00}\right)}^{3/2}}E\sqrt{\frac{g_{00}g_{\phi \phi }-g_{0\phi }^{2}}{g_{00}L^{2}-2g_{0\phi }LE+g_{\phi \phi }E^{2}}}$$

## 4. Kerr–Newman Space-Time

As stated in the Introduction, we start from the empty space-time surrounding a spinning mass. The number of exact solutions of the Einstein equations is quite small, but hopefully we have one precisely in the case of a spinning mass; specifically the central object turns out to be a rotating black hole with a quasi-spherical event horizon. We are of course referring to the Kerr solution [19].

In order to be a bit more general we shall include the possibility that the central source be also electrically charged: this is known as the Kerr–Newman [20,21] solution. For completeness we must specify that the Kerr–Newman solution does not include in the Einstein equations a cosmological constant: in practice, it does not consider the presence of dark energy. Here, however, we are not concerned with the cosmic scale.

The properties of such a space-time are synthesized in the line element:

(16) $$\begin{array}{ccc} ds^{2} & = & \frac{r^{2}-2mr+a^{2}+q^{2}-a^{2}\sin^{2}\theta }{r^{2}+a^{2}\cos^{2}\theta }c^{2}dt^{2}+2a\sin^{2}\theta \left(\frac{2mr-q^{2}}{r^{2}+a^{2}\cos^{2}\theta }\right)cdtd\phi \\ & & -\frac{{\left(r^{2}+a^{2}\right)}^{2}-\left(r^{2}-2mr+a^{2}+q^{2}\right)a^{2}\sin^{2}\theta }{r^{2}+a^{2}\cos^{2}\theta }\sin^{2}\theta d\phi^{2}\\ & & -\frac{r^{2}+a^{2}\cos^{2}\theta }{r^{2}-2mr+a^{2}+q^{2}}dr^{2}-\left(r^{2}+a^{2}\cos^{2}\theta \right)d\theta^{2} \end{array}$$

Here we use Boyer–Lindquist coordinates. The meaning of the physical parameters (all lengths) is summarized as follows:

(17) $$m=G\frac{M}{c^{2}};\;a=\frac{J}{Mc};\;q^{2}=\frac{GQ^{2}}{4\pi \varepsilon_{0}c^{4}}$$

*M* is interpreted as the mass of the source, *J* its angular momentum, *Q* its charge; *G* is the Newton constant and $\varepsilon_{0}$ is the dielectric constant of the vacuum.

In order to evidence the travel time asymmetry in a Sagnac-like configuration we may use any “messenger” travelling along a closed path (in space). The simplest is to refer to light so that it is ds=0.

The standard result is the one shown in Equation (8) which, for a circular trajectory in the equatorial plane, coincides with Equation (12).

The sign of the tof difference depends on which sense we assume to be positive, so it is irrelevant provided the next steps be consistent with the initial choice. For our purposes and without reduction of generality we may choose a closed circuit in the equatorial plane (θ=π/2) and at a constant radius (r=R), i.e., a circle. Under these conditions formula (12) holds and the result is:

(18) $$\Delta T=\frac{4\pi }{c}\frac{g_{0\phi }}{g_{00}}=\frac{4\pi }{c}a\frac{2mR-q^{2}}{R^{2}-2mR+q^{2}}$$

Coordinate time coincides with the time of a distant observer at rest with the mass in the origin. Space-time surrounding such an observer is flat: we may verify it letting *r* go to ∞ in the line element of Equation (16). The metric tensor there is asymptotically diagonal, so, should the closed path of light be entirely located in those far regions (R→∞), the tof asymmetry would also tend to zero: result emerging from Equation (18).

Still using coordinate time, but getting closer and closer to the central mass we may see what happens at the other end of the radial distances, i.e., for R→0. Remarkably the tof asymmetry is finite and non-zero:

(19) $${\left.\Delta T\right|}_{R=0}=\frac{4\pi }{c}a=4\pi \frac{J}{Mc^{2}}$$

By the way, if the closed path revolves around the axis of the central mass, but is not contained in the equatorial plane, the result is different. Considering a circular circuit in the plane $r\cos\theta =z_{0}$ constant, the time asymmetry is

(20) $${\left.\Delta T\right|}_{R\cos\Theta =z_{0}}=-\frac{4\pi }{c}\frac{a\left(2mR-q^{2}\right)\sin^{2}\Theta }{R^{2}-2mR+a^{2}+q^{2}-a^{2}\sin^{2}\Theta }$$

Now Θ is the half-aperture of a cone having its vertex in the origin. Introducing the variable ρ=RsinΘ such that $R=\sqrt{\rho^{2}+z_{0}^{2}}$ (20) becomes

$${\left.\Delta T\right|}_{R\cos\Theta =z_{0}}=-\frac{4\pi }{c}\frac{a\left(2m\sqrt{\rho^{2}+z_{0}^{2}}-q^{2}\right)\frac{\rho^{2}}{\rho^{2}+z_{0}^{2}}}{\rho^{2}+z_{0}^{2}-2m\sqrt{\rho^{2}+z_{0}^{2}}+a^{2}+q^{2}-a^{2}\frac{\rho^{2}}{\rho^{2}+z_{0}^{2}}}$$

and letting ρ go to 0, i.e., reducing the circuit to the axis above or under the origin, we see that

$${\left.\Delta T\right|}_{\rho \to 0}=0$$

The asymmetry disappears.

It is only in the origin, where the source is located, that the asymmetry is the non-zero (19). Being at a point, this difference may be read as a time uncertainty there.

We may think to apply this result to an electron, so that the eigenvalue of the axial component of the angular momentum (which is the semiclassical interpretation of the spin) is J=ℏ/2. Equation (19) now reads:

(21) $$\Delta T=\frac{h}{Mc^{2}}$$

Considering that $Mc^{2}$ is the relativistic (rest) energy of the particle, (21) coincides with the lower limit of the Heisenberg uncertainty formula. Remarkably, the result does not depend neither on *G* nor on *q*: the charge looses any role and even the coupling of the mass to the curvature of space-time disappears. What matters is not the curvature but only the chiral symmetry of space-time induced by the rotation of the mass.

We have made reference to an electron, both because it is a Fermion and because it is a lepton, then has no internal structure; but, should we have considered a proton the result would have been the same, and, apart for a factor of the order of 2, also bosons would have been OK.

The result we have found holds for a distant observer, not directly affected by the curvature and peculiar symmetry of space-time. If instead we look at a local observer orbiting the central mass and getting closer and closer to it, the formula to be used is (15) and for this observer the time of flight asymmetry in the origin falls to zero. The details of this calculation are developed in the Appendix A.

## 5. Concluding Remarks

Our aim, in this paper, was simply to evidence an unexpected link between classical GR formulae and a fundamental relation of quantum mechanics as the Heisenberg uncertainty principle is. As we have seen, the mentioned link appears when considering the asymmetry in the times of flight of light beams coming back to the observer after travelling in opposite directions along the same closed contour (actually the result holds also for other messengers, provided their local velocity along the trajectory depends only on the position and not on the rotation sense). The interesting result emerges when the space-time of a steadily spinning mass is considered and the closed path shrinks to the origin where the central source of gravity is located. In order to be able to call in the quantum spin of a particle we have used an explicit and exact solution of the Einstein equations, the Kerr–Newman solution, assuming that the central source be an elementary Fermion (a lepton, like an electron, without more internal degrees of freedom). Under these conditions the difference between the times of flight in different rotation senses reduce to an uncertainty on the time measured at the origin; identifying the classical angular momentum of the source with the spin of the Fermion and the central mass with the rest mass of the particle, coinciding with its energy modulo $c^{2}$, we have obtained the lowest value of the Heisenberg formula for energy and time uncertainties. The result does not depend neither on the constant of gravity *G* nor on the charge of the particle, so we may interpret it as a property of the symmetry of space-time when it couples to elementary spinning sources.

We think this result may be inspiring for further work, requiring deeper and more detailed elaborations leading along a path that promises more surprises.

## Data Availability

Not applicable.

## Appendix A. Asymmetry for Orbiting Observers and Clock Effect

For completeness we must recall that the calculation leading to Equation (21) holds for coordinate time and a non-rotating observer, i.e., for an inertial observer located far away. If we look at the situation from the viewpoint of an observer orbiting the central mass at a finite distance from the origin and in the equatorial plane, the formula to be used is (15), i.e., explicitly

(A1) $$\Delta T_{\Omega }=\frac{4\pi }{c}E\frac{aR^{2}\left(2mR-q^{2}\right)}{{\left(q^{2}+R^{2}-2mR\right)}^{\frac{3}{2}}}\sqrt{F_{0}}$$

with

(A2) $$F_{0}=\frac{-a^{2}-q^{2}-R^{2}+2mR}{\left(R^{2}-2mR+q^{2}\right)L^{2}-2\left(2mR-q^{2}\right)aLE-\left(2ma^{2}R+R^{4}-a^{2}q^{2}+a^{2}R^{2}\right)E^{2}}$$

The result now tends to 0 both for R→0 and for R→∞.

Considering the clock effect, the basic formula comes from (11) and is:

(A3) $$\Delta T_{C}=\frac{2\pi }{\omega_{+}-\omega_{-}}\left(\sqrt{g_{00}+2g_{0\phi }\frac{\omega_{+}}{c}+g_{\phi \phi }\frac{\omega_{+}^{2}}{c^{2}}}-\sqrt{g_{00}+2g_{0\phi }\frac{\omega_{-}}{c}+g_{\phi \phi }\frac{\omega_{-}^{2}}{c^{2}}}\right)$$

where now $\omega_{+}$ and $\omega_{-}$ are the free fall angular velocities in opposite directions along the same orbit. Let us say that one corresponds to $L=L_{+}>0$ and the other to $L_{-}=-L_{+}<0$. Recalling Equation (14) it is

$$\left\{\begin{array}{c} \frac{\omega_{+}}{c}=\frac{Lg_{00}-Eg_{0\phi }}{Eg_{\phi \phi }-Lg_{0\phi }}\\ \frac{\omega_{-}}{c}=-\frac{Lg_{00}+Eg_{0\phi }}{Eg_{\phi \phi }+Lg_{0\phi }} \end{array}\right.$$

Let us introduce these value into Equation (A3):

$$\Delta T_{C}=\frac{\pi }{c}\frac{\left(g_{\phi \phi }^{2}E^{2}-L^{2}g_{0\phi }^{2}\right)}{EL\left(Lg_{0\phi }-Eg_{\phi \phi }\right)}\frac{\sqrt{2g_{0\phi }LE-g_{00}L^{2}-g_{\phi \phi }E^{2}}-\sqrt{-\left(g_{00}L^{2}+2g_{0\phi }LE+g_{\phi \phi }E^{2}\right)}}{\sqrt{\left(g_{0\phi }^{2}-g_{00}g_{\phi \phi }\right)}}$$

which in the case of a Kerr–Newman space-time becomes

$$\Delta T_{C}=\frac{\pi }{cEL}\frac{\left(ER^{4}+Ea^{2}R^{2}+2mEa^{2}R-2LmaR+Laq^{2}-Ea^{2}q^{2}\right)}{R^{3}}\frac{F_{1}-F_{2}}{\sqrt{a^{2}+q^{2}+R^{2}-2mR}}$$

where

$$F_{1}=\sqrt{2\left(2mR-q^{2}\right)aLE-\left(R^{2}-2mR^{2}+q^{2}\right)L^{2}+\left(R^{4}+a^{2}R^{2}+2ma^{2}R-a^{2}q^{2}\right)E^{2}}$$

and

$$F_{2}=\sqrt{-\left(\left(R^{2}-2mR^{2}+q^{2}\right)L^{2}+2\left(2mR-q^{2}\right)aLE-\left(R^{4}+a^{2}R^{2}+2ma^{2}R-a^{2}q^{2}\right)E^{2}\right)}$$

When two counter-orbiting identical clocks are compared in the same position along their circular and common orbit they appear to have lost synchrony by the amount $\Delta T_{C}$ per turn. The asynchrony disappears when a=0, i.e., when the source of gravity is not spinning.

We also see that $\Delta T_{C}$ becomes 0 at the horizon $R=q^{2}/2m$ and is of course 0 for R→∞.
